# Facial asymmetry assessment in adults using three-dimensional surface imaging

**DOI:** 10.1186/s40510-015-0106-9

**Published:** 2015-10-21

**Authors:** Arti Patel, Syed Mohammed Shamsul Islam, Kevin Murray, Mithran S. Goonewardene

**Affiliations:** Department of Orthodontics, School of Dentistry, The University of Western Australia, 35, Stirling Hwy, Crawley, Western Australia 6009 Australia; School of Mathematics and Statistics, The University of Western Australia, 35, Stirling Hwy, Crawley, Western Australia 6009 Australia

**Keywords:** Facial aesthetics, Facial soft-tissue asymmetry, 3D surface imaging, Landmark-independent analysis, RMS distance measure, Weibull distribution

## Abstract

**Background:**

The use of three-dimensional (3D) surface imaging is becoming more popular and accepted in the fields of Medicine and Dentistry. The present study aims to develop a technique to automatically localise and quantify soft-tissue asymmetry in adults using 3D facial scans. This may be applied as a diagnostic tool to monitor growth and dynamic changes and to evaluate treatment outcomes.

**Methods:**

3D facial surface data were captured from 55 adults comprising 28 symmetrical faces and 27 asymmetrical faces using a 3dMDface system. A landmark-independent method, which compared the original and the mirrored 3D facial data, was developed to quantify the asymmetry. A Weibull distribution-based probabilistic model was generated from the root-mean-square (RMS) error data for the symmetrical group to designate a level of asymmetry which represented a normal range.

**Results:**

Statistically significant (*p* < 0.0001) differences in the RMS error values were found when comparing symmetrical with asymmetrical groups and a similarly significant difference was identified between the lower and the upper face of the asymmetrical group.

**Conclusions:**

The proposed 3D imaging-based method of identifying and quantifying facial soft-tissue asymmetry was fast and effective. The Weibull distribution-based comparison of a person’s asymmetry with respect to a large sample of symmetrical faces may also be used to evaluate growth, soft-tissue compensations and surgical outcomes.

## Background

Symmetry is a common occurrence in nature. It is defined as “equality or correspondence in the form of parts distributed around a centre or an axis, at the two extremes or poles, or in opposite sides of the body” [1]. Although a mild degree of asymmetry is common in the face of normal human individuals [2–7], orthodontists and surgeons often encounter patients with severe asymmetries. Facial symmetry is historically associated with attractiveness [8–11], and a severe asymmetry may have a psychosocial impact [12]. Severe asymmetries combined with other skeletal deformities may require surgical intervention [13]. Therefore, accurate localisation and quantification of the extent of facial asymmetry are crucial in order to facilitate orthodontic diagnosis and establish treatment goals.

According to Proffit and Severt [14], the prevalence of asymmetry is dependent on the type of presenting malocclusion. Asymmetry was found to be present in 28 % of class II subjects, and a prevalence of 40 % was noted in others (class III patients, patients with a long face and class I). However, the most severe asymmetries are usually associated with craniofacial syndromes such as hemifacial microsomia, clefting anomalies and craniosynostoses [15, 16]. Skeletal asymmetries not linked to craniofacial syndromes are considered related to asymmetrical skeletal development of individual craniofacial structures. This may include asymmetry in the position of the glenoid fossa or asymmetrical development of the mandible. Trauma and infection in the condylar region may also result in the development of asymmetry and perhaps ankylosis of the temporomandibular joint leading to a secondary functional impairment. This may have a profound impact on future growth [17].

The surface of the face is the most visible area that clinicians and lay people appreciate and often forms the basis of aesthetic judgments. Since there is greater emphasis placed on the soft tissues, it is important to have reliable data on the external soft-tissue integument and its balance with the underlying hard-tissue skeleton.

Currently, there are several available methods for capturing and quantifying craniofacial surface morphology. These include direct anthropometry and digital photography as well as newer three-dimensional (3D) surface imaging systems. 3D imaging-based methods of measuring facial asymmetry have become popular and have created a virtual reality paradigm. In addition, 3D image analysis methods have greatly assisted in reducing the magnification errors produced from geometric distortions that commonly affect conventional 2D-acquisition methods. The use of 2D projections, which aim to quantify 3D asymmetric objects, introduces inaccuracies [18] because reference points are imprecise. In a recent report which assessed ten human skulls, de Moraes et al. found poor reproducibility of reference points in digital 2D images and the true physical measurements (kappa = 0.609) and an almost perfect agreement (kappa = 0.92) when 3D data were analysed [19]. Cheung et al. and Tai et al. [20, 21] also reported similar and supportive results. From an anthropometric perspective, 3D surface capture has many additional advantages over traditional methods. Craniofacial angles, surface areas and volumes may be quantified along with linear distances. The extraction of *X*, *Y* and *Z* coordinate data, as well as the generation of a permanent archival record of a subject’s face, has been made possible [18, 20–22].

Previous reports have indicated that asymmetry largely exists in the lower third of the face [14, 23–25]. However, most studies have focused on hard-tissue asymmetry. Further information related to the dominance of soft-tissue asymmetry in the facial thirds is needed, particularly since it has been demonstrated that the nasal region is more critically evaluated as an observer’s focus is initially directed to the central region of the face [26].

In addition to a pretreatment morphological assessment and quantification of soft tissue, 3D soft-tissue scanning may be useful for assessing the impact of treatment in the management of asymmetry by appropriately developed superimposition techniques [27]. Furthermore, it may be possible and advantageous to monitor growth or treatment-induced changes in three dimensions.

The aim of the present study was to devise a relatively quick, simple and landmark-independent 3D imaging-based technique that might potentially be valuable as a diagnostic tool for orthognathic and cosmetic surgical planning for patients with soft-tissue asymmetry. Moreover, it was expected that the technique would establish a threshold, above and below which asymmetry might be identified in a region-specific analysis.

## Methods

Ethics approval to conduct this study was obtained from the Human Research Ethics Committee of The University of Western Australia. All assessments were performed in accordance with the guidelines of the National Health and Medical Research Council of Australia.

The study was a retrospective evaluation of two groups of male and female subjects, 20 years of age and older who were treated in the Dental School Clinic and a Private Orthodontic Practice in Perth, Western Australia. The first group was comprised of 27 patients who presented for the management of an obvious facial asymmetry. The second group was formed with 28 subjects with no apparent facial asymmetry. Appropriate group allocation was confirmed by three different orthodontists on separate occasions based on the comprehensive evaluation of the facial radiographs and photographs collected as part of the subjects’ treatment records. The subjects in the first group were a combination of different angle classifications (classes I, II or III), and all of these patients required combined orthognathic surgery to correct their asymmetry. The subjects in the second group were all angle class I subjects. Patients who presented with a craniofacial deformity or syndrome were not included in any of the groups.

**Fig. 1:**
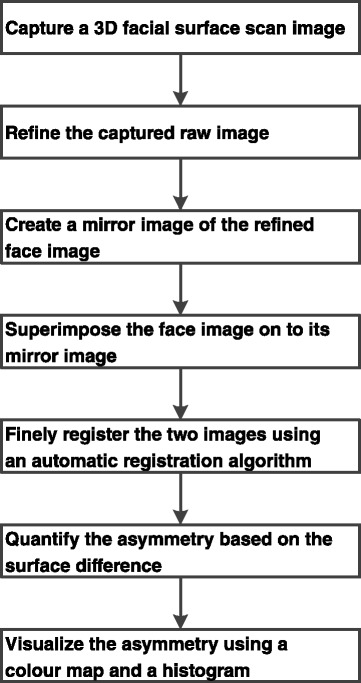
Basic block diagram of the proposed approach for facial asymmetry analysis

The method of the facial asymmetry analysis is illustrated in Fig. 1. Initially, each subject was scanned using a 3dMDface scanning system (3dMD Inc., Atlanta, GA, USA). The scanner incorporated a multi-camera configuration mounted on a wall. Three cameras were arranged on each side of the subject who was seated on an adjustable chair and asked to adopt a natural head posture by looking at a specific point marked on an opposing wall. Natural head posture was adopted for this study as it has been shown to be clinically reproducible [28-30]. The subjects were also required to keep their jaws in a relaxed state just before the images were taken. If the subject moved during scanning, the procedure was repeated.

The 3dMDface scanning system scanned a subject’s head and neck area (a 180° facial image from ear to ear) in 1.5 ms at the highest resolution available (1236 × 1624 pixels). The two acquired stereo camera viewpoints (captured by the cameras located on two sides) were combined using a stereophotogrammetry technique which produced a single 3D image.

The scanned images were refined on a personal standard desktop computer using the 3dMDpatient software (3dMD Inc., Atlanta, GA). The software provided automatic tools for removing ‘defects’ such as spikes or holes in the point cloud generated by the scanner. A cylindrical area of the face extending from ear to ear was cropped to eliminate the shoulders and neck.

**Fig. 2:**
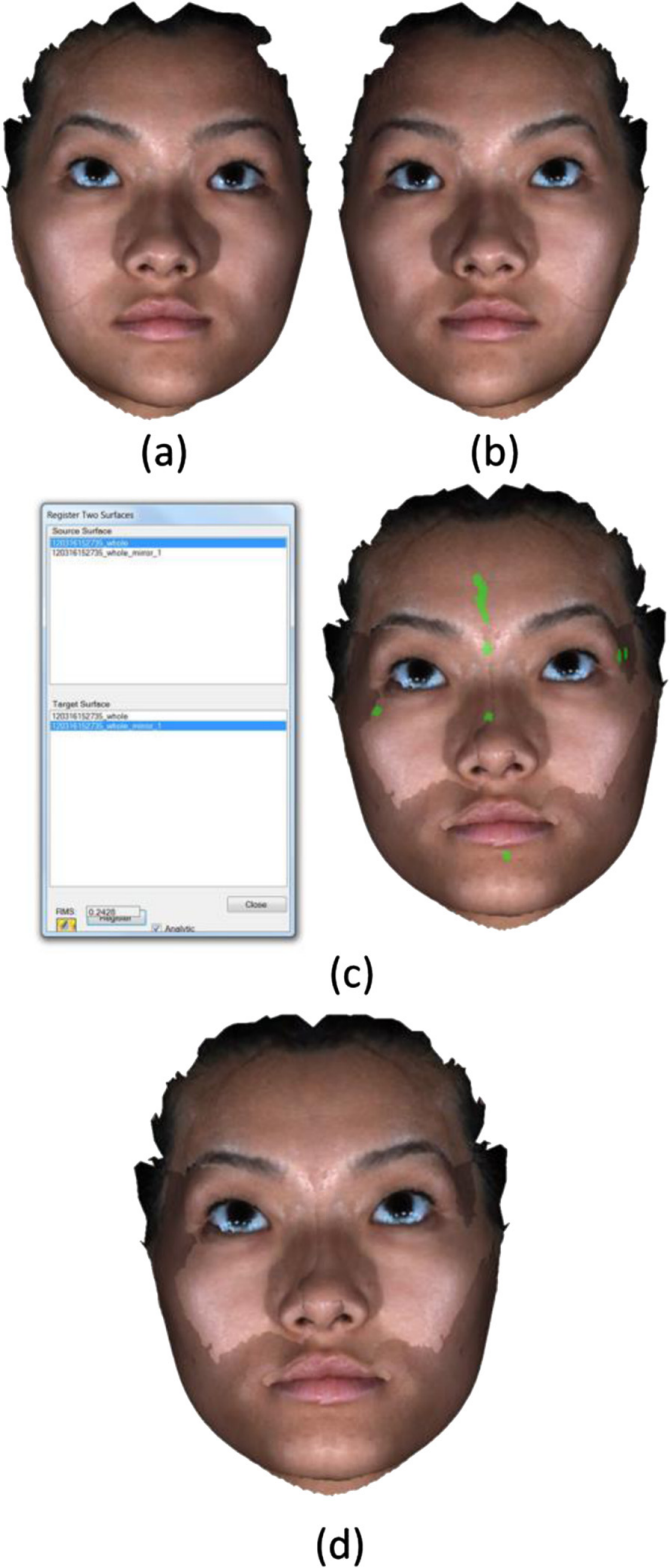
Image registration process. **a** Original image. **b** Mirror image of **a** constructed along an arbitrary plane. **c** Initial registration based on some selected regions (shown with *green colour*). **d** The fine registration of the two images using that Levenberg-Marquardt algorithm

The quantification of facial asymmetry was performed on each patient’s 3D data using the 3dMDvultus software (3dMD Inc., Atlanta, GA) by superimposing an image onto its mirror image, similar to the approach described by Nonda et al. [27]. The mirror image was created, in accordance with Cevidanes et al. [31] by reflecting along an arbitrary plane outside of the face (Fig. 2). Subsequently, the original and the mirror images were aligned with respect to surface features of the forehead, over the root of the nose and zygoma. Following manual registration, an automatic fine registration was performed using the Levenberg-Marquardt algorithm [32] carried out to an error level below 0.5.

**Fig. 3:**
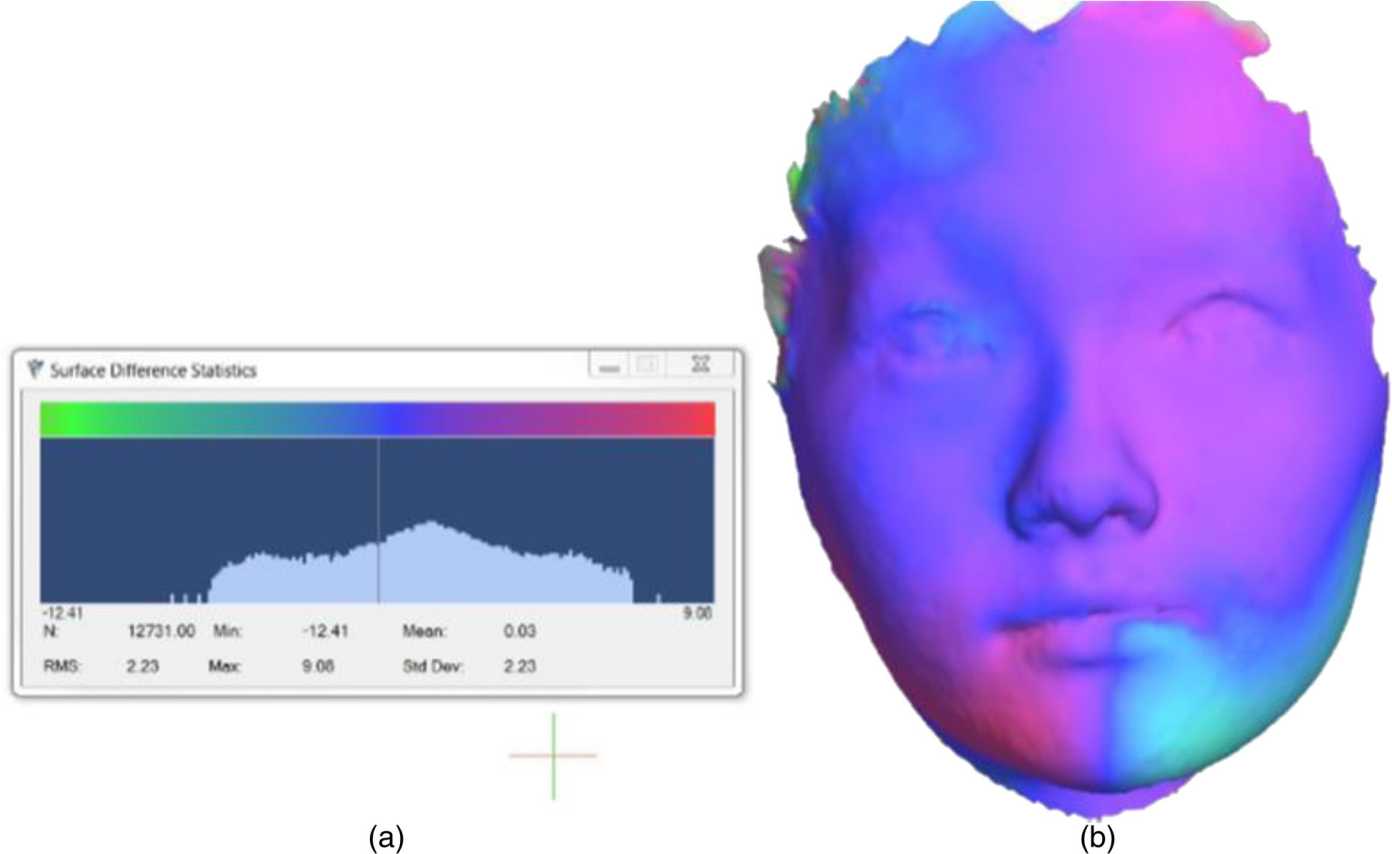
Quantification and visualisation of facial asymmetry. **a** Statistics of the difference of two registered facial surfaces (original and mirror). **b** Registered face images with *colour map*

The distances between the data points of the two registered surfaces were colour-coded for the purpose of asymmetry visualisation. Every data point of the original surface image was assigned a colour based on its distance from the corresponding data point in the mirrored surface image. A histogram (colour-coded scale), corresponding to the distances, was also generated as illustrated in Fig. 3. Colours at the positive end of the histogram depicted regions which had outward movement or convexity with respect to the reference (original) surface. Colours at the negative end represented regions which had inward movement or concavity with respect to the reference surface. Colours at the middle of the histogram highlighted regions which had almost no differences between the two superimposed surfaces. The parameters noted in the histogram were defined as follows:

- *N—*number of data points in the selected region
- RMS (root-mean-square)—the square root of the mean of the squares of the discrete values
- Min (minimum)—the smallest area of deviation between the two selected surfaces displayed in millimetres
- Max (maximum)—he largest area of deviation between the two selected surfaces displayed in millimetres
- Std dev—the standard deviation of the negative and positive values over the selected region

Minimum and maximum measurements are found to be greatly influenced by the boarder of the images (especially the hairline) and by the changes near the eyes (due to blinking) or mouth (due to lip posture) which may result in arbitrary minimum and maximum measurements. Therefore, these two measurements are discarded in the analysis.

**Fig. 4:**
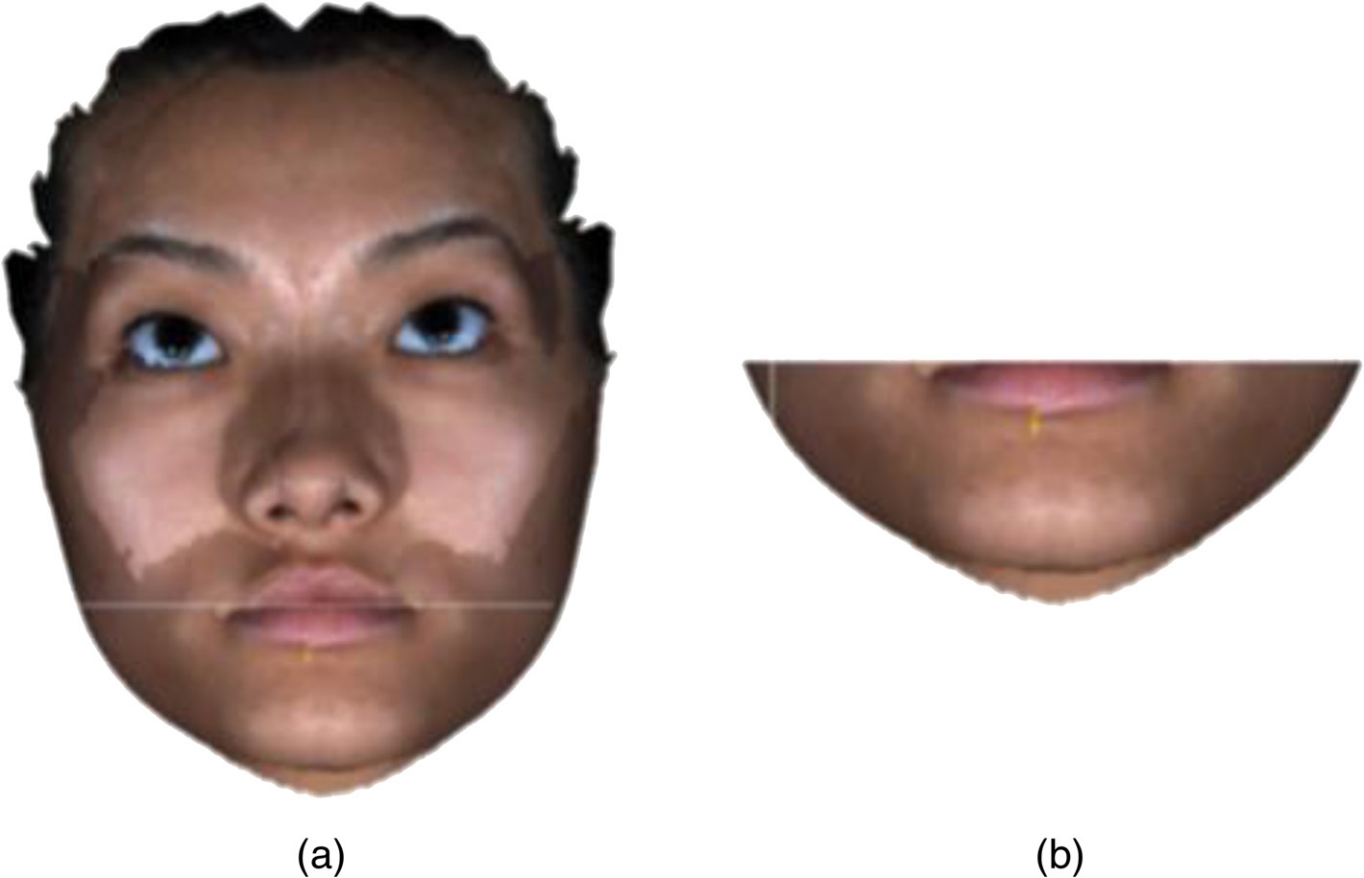
Segmentation of the lower third of the face. **a** *Line* along which the lower face was segmented. **b** Lower face after segmentation

The middle and lower facial thirds were separated by horizontal lines running through the outer canthus of the eye to the outer commissure of the lips (upper third) and below the outer commissure of the lips (lower third) as illustrated in Fig. 4. This separation was performed after the registration of the original and mirror whole face images. Colour maps were also generated for the lower and middle third of the faces to visualise the asymmetry in those regions.

### Statistical analysis

A linear mixed model approach was used to determine the impact of the fixed factors of location (whole, mid or lower face) and symmetry (asymmetrical or symmetrical subjects), on the response variables of the RMS, minimum, maximum and mean. A variance stabilising log transformation was used for the RMS. Additionally, given the skewed nature of the whole-face RMS measurements, a two-parameter Weibull distribution was fitted to the data on the 28 symmetrical subjects, using methods of maximum likelihood to estimate the parameters from the data.

To investigate the intra-observer reliability, the 3D data of five randomly selected subjects were analysed for a second time 2 weeks later and intraclass correlation coefficients (ICC) were estimated. The interpretation of the ICCs was based on a sliding scale characterised by Landis and Koch [33], indicating values <0 as no agreement, 0–20 as slight, 21–40 as fair, 41–60 as moderate, 61–80 as substantial and 81–100 as almost perfect agreement. All statistical analyses were performed using R environment for statistical computing [34].

## Results

The Intraclass Correlation Coefficients (ICC) were calculated by face type (asymmetrical or symmetrical), location (upper or lower face) and type of measurement (RMS error, mean, minimum, and maximum). Apart from a couple of measures, the ICCs were generally high.

**Fig. 5:**
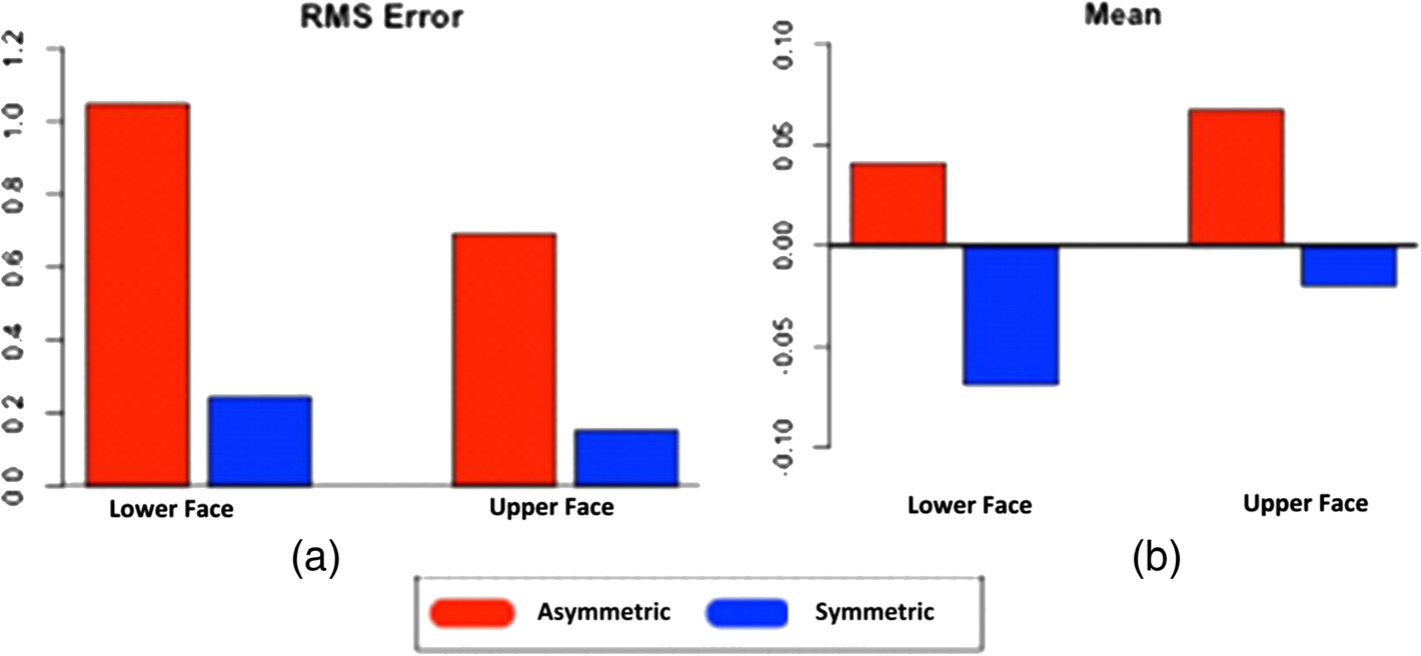
The changes in the upper and lower faces of asymmetrical and symmetrical subjects measured in **a** RMS **b** mean values

Table 1 shows the summary statistics for the different measurements of the upper, lower and whole face in the 27 asymmetrical and 28 symmetrical subjects, whilst Table 2 and Fig. 5 provide comparisons of upper/lower face and asymmetry/symmetry combinations separately for each of the measurements (RMS error and mean value). On average, the RMS error was higher in the asymmetrical groups compared with the symmetrical groups when considering all three locations (lower, upper and whole faces) separately (3.33, 2.39 and 2.85 versus 1.37, 1.23 and 1.52, respectively). Upon comparison of the lower face in the asymmetrical subjects against the lower face in the symmetrical subjects, the difference in the RMS error values was statistically significant (*p* < 0.0001). Similarly, a statistically significant difference in the RMS error values (*p* < 0.0001) can be seen in comparison of the lower face and upper face in the asymmetrical subjects and the upper face in the asymmetrical subjects against the upper face in the symmetrical subjects (*p* < 0.0001). No statistically significant differences (*p* = 0.2505) were observed when comparing the lower and upper face in the symmetrical subjects.

**Table 1:** Summary statistics for different measurements on the upper, lower and whole faces in the 27 asymmetrical and 28 symmetrical subjects

Measurements		Asymmetrical			Symmetrical			Total
		Lower	Upper	Whole	Lower	Upper	Whole	
RMS	Average	3.33	2.39	2.85	1.37	1.23	1.52	2.10
	Std dev	1.82	1.88	1.54	0.52	0.42	0.39	1.48
Mean	Average	0.04	0.07	0.06	−0.07	−0.02	−0.00	0.01
	Std dev	0.43	0.25	0.30	0.29	0.14	0.19	0.28

**Table 2:** Statistical differences in different measurements upon comparison of the upper and lower faces in the asymmetrical and symmetrical subjects

Pairwise comparison	Measurements	Estimated value	Std err	*T* value	*p* value
Lower asymmetrical vs. lower symmetrical	RMS	0.8033	0.1273	6.31	<0.0001*
	Mean	0.1097	0.07940	1.38	0.1711
Lower asymmetrical vs. upper asymmetrical	RMS	0.3563	0.08098	4.40	<0.0001*
	Mean	−0.02630	0.05130	−0.51	0.6104
Lower symmetrical vs. upper symmetrical	RMS	0.09239	0.07952	1.16	0.2505
	Mean	−0.04893	0.05038	−0.97	0.3358
Upper asymmetrical vs. upper symmetrical	RMS	0.5394	0.1273	4.24	<0.0001*
	Mean	0.08704	0.07940	1.10	0.2763

*Statistically significant differences

On average, the mean value measurements were higher in the asymmetrical groups compared with the symmetrical groups at the three locations (lower, upper and whole faces) separately (0.04, 0.07 and 0.06 versus −0.07, −0.02 and −0.00, respectively). Although the summary statistics indicated variation in the asymmetrical and symmetrical subjects in the mean value measurements, the comparative data indicated no statistically significant differences between all combinations in facial symmetry (asymmetrical versus symmetrical) and location (upper and lower faces).

**Fig. 6:**
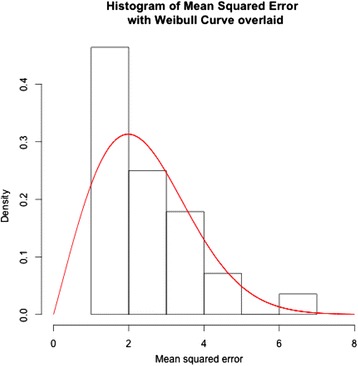
A Weibull graph created from a histogram of the whole face in the symmetrical subject group

**Fig. 7:**
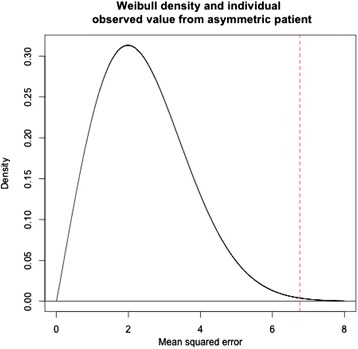
An illustration of an example on how to utilise the Weibull distribution graph created from a dataset of symmetrical subjects. The *dotted line* illustrates a patient that lies in the 99.8th percentile

Figure 6 illustrates a two-parameter (shape and scale) *Weibull distribution* fitted to the RMS error data for the whole face in the 28 symmetrical subjects. The Weibull curve is superimposed over the histogram and thus provides a percentile measurement for any given individual. Therefore, this curve can be used to predict the severity of asymmetry in a given subject. For example, as illustrated in Fig. 7, an asymmetrical subject with an observed RMS measurement of 2.6 (the square of which is 6.76) falls in the 99.8th percentile (represented by the red dotted line). This percentile value indicates 99.8 % of the symmetrical patients (according to this group) would have a value less than the observed RMS measurement value for this patient. Since a large RMS value is proportional to the level of asymmetry, the extreme RMS value potentially indicates that this particular patient is unlikely to be from the symmetrical distribution.

## Discussion

The development and advancement of technology has improved diagnosis and treatment planning for significant dentofacial deformities [35]. Peck et al. [36] demonstrated that the extent of skeletal asymmetry was confounded by significant deviations in overlying soft-tissue dimensions. Therefore, correction of the skeletal and dental asymmetry may not result in a symmetrical aesthetic soft-tissue form.

Historically, a 2D analysis of asymmetry with facial photographs has complemented a clinical examination using linear and planar differences between each side of the face which are prone to measurement error [19]. With the advancement of 3D technology, cost effective, fast, reliable, accurate and noninvasive methods have been developed to provide 3D scanning of facial soft tissues. Since the 3D facial analysis methods described in the present study are independent of the 3D image capturing system, any 3D facial scanner may be used for acquiring 3D facial surface data.

In the present study, the quantitative analysis of facial asymmetry was performed by measuring the distances between the original and mirror images of a subject. It was observed that the RMS errors between the two sides of the face were higher for asymmetrical compared with symmetrical faces regardless of the location (upper and lower) (Table 1 and Fig. 5). This implied that an asymmetrical face, on average, has a wider range of points (minimum to maximum) in comparison with symmetrical faces.

The statistical differences in the RMS error measurements (Table 2) indicated highly significant differences for the upper and lower faces independently between the asymmetrical and symmetrical subjects. A statistically significant difference in the RMS values in the upper and lower face in the asymmetrical subjects was observed. This finding matches those of previous studies which indicated facial asymmetry predominated in the lower third of the face [3, 23–25]. However, similar to Djordjevic et al. [37], no statistically significant difference was identified in symmetrical subjects.

The mean value analysis calculated the mean of the number of data points in a selected region. The mean value measurements were higher in the asymmetrical groups compared with the symmetrical groups at the three locations (lower, upper and whole faces) separately (Table 1 and Fig. 5). A comparison of the mean difference data (Table 2) indicated no statistically significant effects for all combinations in facial asymmetry (asymmetrical versus symmetrical) and location (upper versus lower face). This may be explained by a subject who is asymmetrical would be expected to have a wide spread in minimum and maximum values, and hence, the mean value could potentially be zero. In addition, it would be expected that a smaller spread in the minimum and maximum values would occur in symmetrical subjects and hence, still result in a mean value of zero.

One of the strengths of the present study is that a face was only compared with its mirror image which avoided the problem of assessing faces of different sizes. Using an arbitrary plane outside of the face eliminated the problem of identifying a midsagittal plane which is difficult to define, especially in patients with asymmetry [38]. Moreover, the colour maps (Fig. 3) obtained by the superimposition (best fit registration) of the original and mirror facial images provided a detailed quantitative assessment of asymmetry using all of the available facial points instead of a limited number of facial landmarks. This also prevented inaccuracies associated with individual landmark identification. The number of registered points relied on the size of the superimposed area and the proportion of asymmetrical facial regions. Therefore, two surfaces were first registered based on the entire face and then segmented into lower and middle thirds for a more in-depth assessment of asymmetry of specific regions.

It is important to select the most appropriate reference region when manually superimposing the 3D images. The ideal reference region should be a stable area such as the forehead [39] which is unlikely to be affected by surgical intervention. Some rotational errors were encountered during forehead registration as the forehead is relatively flat and lacks unique 3D shape features. Therefore, similar to Jayaratne et al. [40], surfaces over the root of the nose and zygoma were chosen. The relatively thin and immobile nature of the overlying soft tissues in these regions helped eliminate some of the spatial image malalignments. Guest et al. [41] evaluated four methods of superimposition in two surgical patients. All methods were found to produce errors in the calculation of final surface changes. However, the registration algorithm chosen in the present study produced promising and realistic results because no assumptions were made about the direction of displacement between surfaces.

The Weibull distribution-based probabilistic model illustrated in Figs. 6 and 7 was generated from a data set of 28 symmetrical subjects. Therefore, it may be too simplistic to make a clinical recommendation regarding the use of this curve. However, as this curve represents the quantification of symmetry distribution, it may be a useful diagnostic tool in identifying asymmetries not immediately recognised by the naked eye. It may also enable the clinician or researcher to monitor the progress of diagnosed asymmetries, if they are progressive. The probabilistic model may be useful in the identification of significant deviation beyond a particular threshold. An individual can have their RMS value converted to a corresponding percentile from the Weibull curve, to represent the level of deviation from symmetry, thus potentially providing a reference to evaluate treatment outcomes when asymmetries have been addressed.

Apart from the smaller dataset, the present study has the following limitations. The methodology in selecting an external reference plane and mirror imaging was not fully automated; therefore, minor individual technique differences may account for user-related error. It would also be interesting to prepare subset dependent on class of malocclusion to determine if there are class-based associations with distribution and magnitude of facial asymmetry. Unfortunately this was not possible with the database currently available but would be considered with subsequent follow-up studies.

## Conclusions

This study presents a method to automatically localise and quantify soft-tissue asymmetry in adults from their 3D facial scans. The method is relatively fast, simple and landmark independent and is a valuable diagnostic and treatment planning tool for orthognathic and cosmetic surgery patients with soft-tissue asymmetry. Our method can enhance clinician-patient communication by identifying areas of deformity, the level of asymmetry and relative relationships between different components of the face and presenting these interactively on a screen in front of the patient. The results of this study suggest that the use of RMS error measurements is predictable and useful as an indication of facial soft-tissue asymmetry. By utilising a Weibull curve in this study, we have identified a prospective means to assess quantitative comparisons of growth, tissue compensation, and surgery outcomes. However, to make better use of the distribution, this probabilistic model needs to be generated from a larger dataset which presents an opportunity for future research.
